# Galectin-3 as a Predictor of Left Ventricular Reverse Remodeling in Recent-Onset Dilated Cardiomyopathy

**DOI:** 10.1155/2018/2958219

**Published:** 2018-06-19

**Authors:** Konstantinos Karatolios, Georgios Chatzis, Volker Holzendorf, Stefan Störk, Anette Richter, Davis Binas, Bernhard Schieffer, Sabine Pankuweit

**Affiliations:** ^1^Department of Cardiology, Angiology and Intensive Care, Philipps University Marburg, Marburg, Germany; ^2^Clinical Trial Center Leipzig, Faculty of Medicine, University of Leipzig, Leipzig, Germany; ^3^Comprehensive Heart Failure Center (CHFC), University and University Hospital Würzburg, Würzburg, Germany

## Abstract

**Objectives:**

Studies have evaluated the association of galectin-3 and outcome in patients with heart failure. However, there is still scarce evidence concerning the clinical usefulness and predictive value of galectin-3 for left ventricular reverse remodeling (LVRR) in patients with recent-onset dilated cardiomyopathy (RODCM).

**Patients and Methods:**

Baseline galectin-3 was measured in 57 patients with RODCM. All patients were followed for at least 12 months. The study end point was LVRR at 12 months, defined as an absolute improvement of the left ventricular ejection fraction of ≥10% to a final value of ≥35%, accompanied by a decrease in the left ventricular end diastolic diameter of at least 10%, as assessed by echocardiography. In receiver operating characteristic curve analysis, the optimum cut-off value for baseline galectin-3 with the highest Youden index was 59 ng/ml.

**Results:**

Overall, LVRR at 12 months was observed in 38 patients (66%). In a univariate analysis, NYHA functional class and baseline galectin-3 levels were associated with LVRR. After adjustment for covariates, galectin-3 remained an independent predictor for LVRR.

**Conclusions:**

Our study suggests that baseline galectin-3 is an independent predictor of LVRR. Low levels of galectin-3 may be regarded a useful biomarker of favorable ventricular remodeling in patients with RODCM.

## 1. Introduction

Dilated cardiomyopathy (DCM) is a major cause of heart failure and currently the leading indication for heart transplantation, with an estimated prevalence of 40 cases per 100000 individuals [1, 2]. Current treatment regimens and their implementation have contributed to improved prognosis in patients with heart failure [3]. Patients with recent-onset dilated cardiomyopathy (RODCM), defined as the duration of heart failure symptoms less than 6 months, have the potential for myocardial recovery as reflected mainly by the left ventricular reverse remodeling (LVRR) [4]. In turn, LVRR, defined as the improvement of the systolic left ventricular ejection fraction (LVEF) and concomitant decrease of left ventricular end diastolic diameter (LVEDD), is associated with a favorable prognosis in patients with DCM [5]. However, prediction of LVRR in clinical practice is challenging and not well delineated. Cardiac biomarkers reflecting different aspects of cardiac pathophysiology have emerged as promising tools not only in diagnosis and monitoring, but also for prediction in a variety of cardiovascular diseases [6].

The novel cardiac biomarker galectin-3 (Gal-3) is a beta galactoside binding lectin involved in fibrogenesis and inflammatory response in the failing heart. Gal-3 is thought to mirror pivotal processes mediating maladaptive cardiac remodeling [7]. Several studies reported on improved risk prediction of mortality and ventricular remodeling using Gal-3 in patients with acute and chronic heart failure of various etiologies [8–12]. Recently, an investigation in 262 patients with nonischemic DCM enrolled in our institution showed that analysis of Gal-3 as a continuous variable had shown significant results neither for the overall DCM cohort nor for any of the subgroups with familial or inflammatory/viral etiology. Nevertheless, in quartile model analysis, Gal-3 was significant for all-cause and cardiac mortality, whereby intermediate values were associated with better outcome [13]. Gal-3, however, may also play an important role in the early phases of heart failure (i.e., reverse remodeling), and therefore, we investigated the performance of Gal-3 to predict LVRR in patients with RODCM. The evidence concerning the clinical usefulness and predictive value of Gal-3 for LVRR in RODCM is still scarce [14].

## 2. Patients and Methods

**Figure 1:**
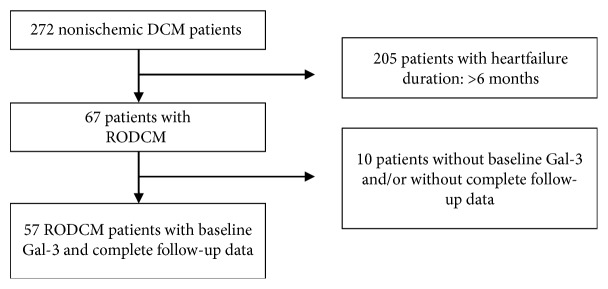
Patients' flow chart. DCM: dilated cardiomyopathy; RODCM: recent-onset dilated cardiomyopathy; Gal-3: galectin-3.

From September 2004 to March 2008, we prospectively enrolled 272 consecutive patients with nonischemic DCM [15, 16]. Patients between 18 and 75 years of age were included if they had a LVEF of <45% and a Henry index of >117% estimated by echocardiography with no evidence of significant valve disease. Coronary artery disease (>50% diameter luminal stenosis in one or more epicardial vessels) was excluded in all patients by means of coronary angiography, and all patients underwent endomyocardial biopsy (EMB). Patients were excluded if they demonstrated one or more of the following parameters: peripartum cardiomyopathy, history of myocardial infarction, systemic hypertension, alcohol abuse, and drug dependency. Out of the 272 patients, 67 (24.6%) patients had RODCM and 57 (85%) patients with RODCM had Gal-3 measured at baseline with complete follow-up data and were thus included in the present analysis (Figure 1). At baseline, all patients underwent clinical assessment, laboratory studies, electrocardiography, and echocardiographic evaluation with 2-dimensional echocardiography. The measurement of variables was based on the harmonized assessment protocol for patients with DCM used within the Competence Network Heart Failure Germany [17]. The diagnosis of DCM was made according to the criteria of the position statement from the European Society of Cardiology working group on myocardial and pericardial diseases [18, 19]. The study was designed as a prospective observational investigation, and all patients were followed for at least 12 months. Follow-up visits included clinical examination, laboratory studies, electrocardiography, and echocardiography. The study end point was LVRR at 12 months.

LVRR was defined as an absolute improvement of LVEF of ≥10% to a final value of ≥35%, accompanied by a decrease in LVEDD of at least 10%, as assessed by echocardiography at 12 months of follow-up. LVEF was assessed by biplane Simpson's rule using manual tracings of digitized images. LVEDD was measured in the parasternal long-axis view.

The study was approved by the local institutional ethics committee, and all patients provided written informed consent.

## 3. Laboratory Methods

Blood samples were collected into serum-separating tubes and were centrifuged and stored in cryotubes at −70°C for later analyses. Gal-3 levels were measured using an enzyme-linked immunosorbent assay kit (BG Medicine, Waltham, USA). Calibration of the assay was performed according to the manufacturer's recommendations, and values were normalized to a standard curve. Glomerular filtration rate (GFR), expressed as ml/min/1.73 m^2^, was estimated using the formula derived from the modification of diet in renal disease (MDRD) study [20].

## 4. Data Analysis

Data are presented as absolute variables and percentages (%) for categorical variables and either median with interquartile range (IQR: 25th–75th percentile) or mean with standard deviation according to the distribution of the variables. Statistical assessment was performed by univariate and multivariable logistic regression analysis. For the logistic regression analysis, we selected parameters with ascertained or potential influence on outcomes in patients with RODCM, including age, gender, NYHA functional class, systolic blood pressure, GFR, myocardial inflammation, and NT-proBNP. In receiver operating characteristic (ROC) curve analysis, the optimum cut-off value with the highest Youden index for Gal-3 was 59 ng/ml [21]. Analyses were performed with R version 3.3.3 (R Core Team).

## 5. Results

Baseline characteristics of the included 57 patients are shown in Table 1. At study entry, the mean age was 48.9 ± 10.5 years, the LVEF was 30.1 ± 8.7%, and the percentage of NYHA functional classes I, II, III, and IV were 9%, 37%, 53%, and 2%, respectively. Myocardial inflammation in EMB was found in 18 (31.6%) patients, and genomes of cardiotropic viruses were detected by PCR in 17 (29.8%) cases (Table 1).

The medical treatment at baseline included angiotensin-converting enzyme inhibitors (ACEI) or angiotensin-receptor blocker (ARB), *β*-blockers, mineralocorticoid receptor antagonists (MRA), glycosides, and diuretics, which were given to 96% (*n* = 55), 93% (*n* = 53), 77% (*n* = 44), 60% (*n* = 34), and 81% (*n* = 46) of patients, respectively. At 12 months, respective proportions were 96%, 100%, 79%, 47%, and 81%. Importantly, there were no differences in heart failure medications in both groups with LVRR present or absent, neither at baseline nor at the 12-month follow-up. In particular, no differences were observed concerning MRA treatment at baseline and follow-up, since aldosterone antagonists may reverse the profibrotic effect of Gal-3 in experimental models [22]. During the follow-up period, no patient died. Overall, LVRR at 12 months was observed in 38 patients (66%), and 19 patients (33%) reached a LVEF of >50%.

### 5.1. Prediction of LVRR from Baseline Parameters

**Figure 2:**
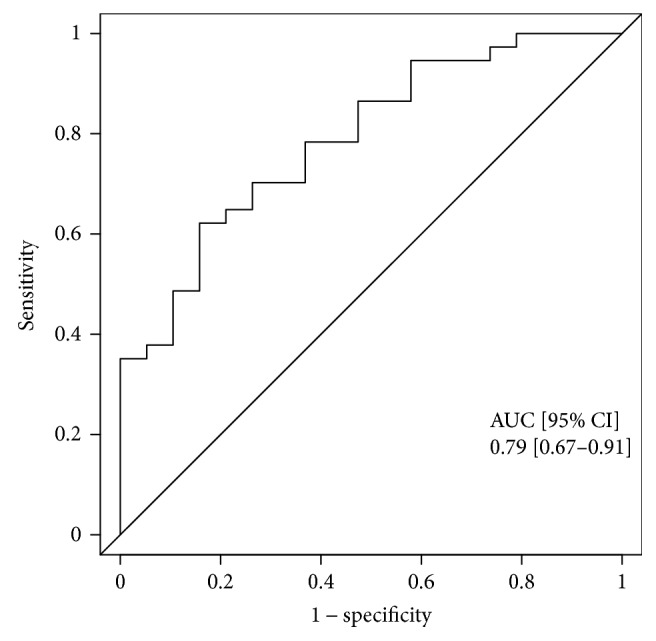
Receiver operating characteristic curve analysis for galectin-3. AUC: area under the curve; CI: confidence interval.

In a univariate analysis, NYHA functional classes I and II [OR 3.46 (95% CI 1.09 to 12.51), *p* = 0.044] and galectin-3 < 59 ng/ml [OR 5.94 (95% CI 1.67 to 23.49), *p* = 0.007] at baseline demonstrated predictive value for LVRR (Table2). After adjustment for the covariates age, gender, NYHA functional class, systolic blood pressure, GFR, myocardial inflammation and NT-proBNP, a Gal-3 level of <59 ng/ml at baseline remained an independent predictor for LVRR [OR 8.88 (95% CI 1.85 to 56.48), *p* = 0.02] (Table 2).  Figure 2 depicts the ROC curve analysis of Gal-3 after the multivariate logistic regression analysis.

## 6. Discussion

In this study, we evaluated the utility of Gal-3 to predict LVRR in patients with RODCM. Baseline Gal-3 levels of <59 mg/ml were significantly associated with LVRR in uni- and multivariable analyses, thus indicating a favorable outcome. Likewise, higher Gal-3 levels predicted absent LVRR, thus enforcing the need to pay more attention to these patients regarding optimization of treatment.

In this regard, Gal-3 has previously been shown to be a prognostic marker of the adverse outcome in patients with acute or chronic heart failure. The DEAL trial [11] and other studies [10] demonstrated that Gal-3 was an independent predictor of mortality risk in patients with moderate to advanced chronic heart failure of both ischemic and nonischemic origin. Gal-3 was also associated with ventricular remodeling and predicted long-term mortality in patients with severe chronic heart failure [8]. In acute heart failure, elevated baseline Gal-3 levels were an independent predictor of short-term mortality [23], whereas the repeated measurement of Gal-3 was a strong predictor for adverse outcome in patients following admission for acute heart failure [9].

Gal-3 has also been associated with outcome and LVRR in patients with valvular heart disease. Preoperative Gal-3 levels were independently associated with LVRR in heart failure patients undergoing surgical mitral valve repair for functional mitral regurgitation [24]. In patients with symptomatic moderate to severe mitral regurgitation undergoing percutaneous mitral valve repair, preinterventional Gal-3 levels were also associated with LVRR and clinical outcome [25]. Moreover, elevated Gal-3 levels were associated with worse outcome in patients with aortic valve stenosis undergoing transcatheter aortic valve implantation [26].

So far, only one study investigated the predictive value of Gal-3 specifically in patients with RODCM [14]. This study was of similar design albeit slightly smaller than ours; it used the same definitions for LVRR, but was based on serial cardiac magnetic resonance (CMR) tomography. In the study by Kubanek et al. [14] baseline Gal-3 levels were also lower in patients with LVRR compared to patients without LVRR, but no statistical significance was apparent between both groups. However, median Gal-3 levels were more than twofold higher in the group without LVRR compared to the patients' group with LVRR [14]. In accordance with this result, mean Gal-3 levels were also twice as high in patients without LVRR compared to patients with LVRR in our investigation. The disparate statistical result may be explained due to our larger sample size and due to differences in the patient characteristics: in our study, GFR was higher in both groups with or without LVRR compared to the former study. Gal-3 levels inversely relate to renal function in patients with heart failure [27]. In addition, the renal function was not associated with LVRR in our investigation, whereas in the study by Kubanek et al. patients with worse renal function had, surprisingly, a higher propensity for better reverse remodeling [14]. Cardiac magnetic resonance provides important information for diagnostic purposes and risk stratification in patients with DCM, including RODCM [14, 28, 29]. In particular, the presence and extent of late gadolinium enhancement (LGE) detected by CMR was inversely correlated with LVRR in patients with DCM [14, 30, 31]. Although the CMR is a fundamental tool for diagnostic purposes and LGE presence, patterns and quantification helps to assess the probability for LVRR, it is not universally available and may not be performed in every patient (e.g., claustrophobia, very obese patients, and metallic implants). Therefore, additional, readily available parameters, such as Gal-3, are useful to aid risk stratification.

The current study demonstrated that Gal-3 levels are associated with LVRR. Cardiac remodeling represents a compensatory mechanism after an initial myocardial insult, leading to left ventricular dysfunction and ultimately heart failure [32]. The association of Gal-3 and ventricular remodeling is plausible since fibrosis and inflammatory responses are pivotal processes in maladaptive cardiac remodeling [7]. Gal-3 acts on fibroblasts and initiates fibrosis. Myocardial expression of Gal-3 was specifically increased in rats that later developed to heart failure [33]. Furthermore, genetic disruption and pharmacologic inhibition of Gal-3 attenuated cardiac fibrosis, left ventricular dysfunction, and subsequent heart failure in a mice model [34].

## 7. Limitations

There are several limitations related to our study. First, the main limitation of our study is the relatively small sample size, which allows only a very limited number of regressors in respective analyses. However, all patients were phenotyped comprehensively and in a very harmonized fashion in accordance with principles mandated by the Competence Network Heart Failure [17]. Second, we measured Gal-3 only at baseline; we were therefore unable to account for Gal-3 changes over time. Third, we did not investigate the MRA treatment effect in relation to Gal-3 levels and ventricular remodeling. However, there were no differences in MRA treatment in both groups (with LVRR present or absent), neither at baseline nor at follow-up.

## 8. Conclusions

Our study suggests that baseline Gal-3 is an independent predictor of LVRR. Low levels of Gal-3 may be regarded a useful biomarker of favorable ventricular remodeling in patients with RODCM.

## Figures and Tables

**Table 1:** Characteristics of the study population.

Characteristic	All patients (*n* = 57)	LVRR present (*n* = 38)	LVRR absent (*n* = 19)	*p* value
Age (years)	48.9 ± 10.5	48.4 ± 11.8	50.1 ± 7.4	0.57
BMI (kg/m^2^)	26.3 ± 4.0	26.1 ± 4.2	26.6 ± 3.7	0.63
Female, *n* (%)	13 (23)	6 (16)	7 (37)	0.10
Diabetes, *n* (%)	7 (12)	6 (16)	1 (5)	0.40
SBP (mmHg)	118.3 ± 15.5	119.9 ± 14.8	115.1 ± 16.9	0.28
DBP (mmHg)	77.6 ± 8.9	78.2 ± 8.6	76.3 ± 9.5	0.45
Heart rate (bpm)	80 ± 20	78 ± 19	84 ± 21	0.28
NYHA functional class, *n* (%)				0.03
(i) I	5 (9)	4 (11)	1 (5)	
(ii) II	21 (37)	17 (45)	4 (21)	
(iii) III	30 (53)	17 (45)	13 (68)	
(iv) IV	1 (2)	—	1 (5)	
Duration of heart failure (months)^#^	1.9 (1.0–3.3)	2.0 (1.0–3.4)	1.8 (1.0–2.7)	0.70
LBBB	39 (68.4)	25 (65.8)	14 (73.7)	0.76
LVEDD (mm)	67.4 ± 7.2	67.2 ± 8.3	67.7 ± 4.4	0.79
LVEF (%)	30.1 ± 8.7	29.6 ± 8.9	31.1 ± 8.2	0.54
Medication at baseline				
(i) ACEI or ARB	55 (96)	36 (95)	19 (100)	0.55
(ii) ACEI or ARB, ≥50% of recommended dose	26 (46)	19 (50)	7 (37)	0.41
(iii) Betablocker	53 (93)	35 (92)	18 (95)	1.00
(iv) Betablocker, ≥50% of recommended dose	21 (37)	14 (37)	7 (37)	1.00
(v) MRA	44 (77)	29 (76)	15 (79)	1.00
(vi) Digitalis	34 (60)	21 (55)	13 (68)	0.40
(vii) Diuretic	46 (81)	29 (76)	17 (89)	0.30
Medication at follow-up				
(i) ACEI or ARB	55 (96)	36 (95)	19 (100)	0.55
(ii) ACEI or ARB, ≥50% of recommended dose	42 (74)	27 (71)	15 (79)	0.75
(iii) Betablocker	57 (100)	38 (100)	19 (100)	1.00
(iv) Betablocker, ≥50% of recommended dose	40 (70)	28 (74)	12 (63)	0.54
(v) MRA	45 (79)	29 (76)	16 (84)	0.73
(vi) Digitalis	27 (47)	18 (47)	9 (47)	1.00
(vii) Diuretic	46 (81)	28 (74)	18 (95)	0.08
ICD at baseline	0	0	0	
ICD at follow-up	6 (11)	6 (16)	0	0.16
CRT at baseline	0	0	0	
CRT at follow-up	8 (14)	3 (8)	5 (26)	0.10
Galectin-3 (ng/ml)	29.4 (16.3–56.2)	22.7 (15.6–45.9)	47.7 (24.2–63.4)	0.03
NT-proBNP (pg/ml)	1308 (585 to 2880)	1220 (554 to 2192)	1818 (724 to 3206)	0.35
Creatinine (mg/dl)	0.94 ± 0.21	0.95 ± 0.22	0.92 ± 0.21	0.97
GFR (ml/min/1.73 m^2^)	89.4 ± 20.4	92 ± 22	84.3 ± 16.2	0.18
Myocardial inflammation^∗^	18 (31.6)	10 (26.3)	8 (42.1)	0.24
Virus-positive genome^∗^	17 (29.8)	12 (31.6)	5 (26.3)	0.77

Values are *n* (%) or mean ± SD or median (interquartile range, IQR) when appropriate. BMI: body mass index; NYHA: New York Heart Association; LBBB: left bundle branch block; SBP: systolic blood pressure; DBP: diastolic blood pressure; LVEF: left ventricular ejection fraction; LVEDD: left ventricular end diastolic diameter; ACEI: angiotensin-converting enzyme inhibitor; ARB: angiotensin receptor blocker; MRA: mineralocorticoid receptor antagonists; ICD: intracardiac cardioverter defibrillator; CRT: cardiac resynchronization therapy; GFR: glomerular filtration rate. ^#^Duration of heart failure symptoms before inclusion; ^∗^detected in endomyocardial biopsies.

**Table 2:** Predictors of left ventricular reverse remodeling (logistic regression analysis).

	Univariate OR [95% CI]	*p*	Multivariable OR [95% CI]	*p*
Galectin-3: <59 ng/ml	5.94 [1.67–23.49]	**0.007**	8.88 [1.85–56.48]	**0.01**
NYHA functional classes I and II	3.46 [1.09–12.51]	**0.044**	2.60 [0.63–12.68]	0.20
Myocardial inflammation in EMB	0.49 [0.15–1.59]	0.23	0.28 [0.06–1.17]	0.09
Male sex	3.11 [0.87–11.59]	0.08	2.46 [0.50–12.62]	0.26
GFR [per 10 ml/min increase]	1.22 [0.92–1.67]	0.18	1.24 [0.86–1.84]	0.26
NT-proBNP [pg/ml—per tenfold increase]	0.50 [0.13–1.72]	0.28	0.80 [0.13–4.55]	0.80
SBP [per 10 mmHg increase]	1.23 [0.86–1.83]	0.27	1.10 [0.68–1.80]	0.70
Age [per 10 years older]	0.85 [0.48–1.45]	0.57	1.31 [0.64–2.74]	0.46

OR: odds ratio; CI: confidence interval; NYHA: New York Heart Association; EMB: endomyocardial biopsy; GFR: glomerular filtration rate; SBP: systolic blood pressure.

## Data Availability

Interested researchers can request the minimal anonymized dataset from the corresponding author if they meet the criteria for access to confidential data.
